# Epibenthic Assessment of a Renewable Tidal Energy Site

**DOI:** 10.1155/2013/906180

**Published:** 2013-02-06

**Authors:** Emma V. Sheehan, Sarah C. Gall, Sophie L. Cousens, Martin J. Attrill

**Affiliations:** Marine Institute, Plymouth University, Marine Building, Drake Circus, Plymouth PL4 8AA, UK

## Abstract

Concern over global climate change as a result of fossil fuel use has resulted in energy production from renewable sources. Marine renewable energy devices provide clean electricity but can also cause physical disturbance to the local environment. There is a considerable paucity of ecological data at potential marine renewable energy sites that is needed to assess potential future impacts and allow optimal siting of devices. Here, we provide a baseline benthic survey for the Big Russel in Guernsey, UK, a potential site for tidal energy development. To assess the suitability of proposed sites for marine renewable energy in the Big Russel and to identify potential control sites, we compared species assemblages and habitat types. This baseline survey can be used to select control habitats to compare and monitor the benthic communities after installation of the device and contribute towards the optimal siting of any future installation.

## 1. Introduction

Widespread concern over global climate change as a result of fossil fuel use has resulted in an increased interest in renewable energy [1]. Wave and tidal energy developments are receiving increased attention despite the technology being less advanced compared to offshore wind technology because it has great potential in countries with suitable conditions [2–4]. Even though the global environmental benefits of renewables are clear, their local impacts must also be quantified, so that future installations can be effectively managed [5].

Often, species assemblages in locations that are suitable for renewable energy installations are not well understood as these high energy environments are difficult and dangerous to study [6]. As the industry is in its infancy, little is also known about the environmental impacts that are likely to result. A marine renewable energy installation can cause physical disturbance during construction, operation, and decommissioning [7]. Inger et al. [5] suggested that the impacts are likely to be both positive and negative. Installations may act as artificial reefs [8] and provide refuge and feeding grounds for marine fauna. Safety exclusion zones surrounding installation sites are likely to exclude benthic trawling and dredging which damage the sea bed [9] and therefore act as *de facto *Marine Protected Areas (MPAs) [5, 10]. Conversely, the infrastructure associated with these developments may entangle marine organisms, create noise and/or cause scouring of the seabed [5, 7, 11]. Guernsey, Channel Islands, UK, has been identified as a candidate area for tidal energy extraction; however, the lack of benthic data in potential tidal energy sites such as Pentland Firth, Scotland, UK [12], and Guernsey, Channel Islands, UK [13], has prompted calls for baseline surveys as a high priority.

**Figure 1:**
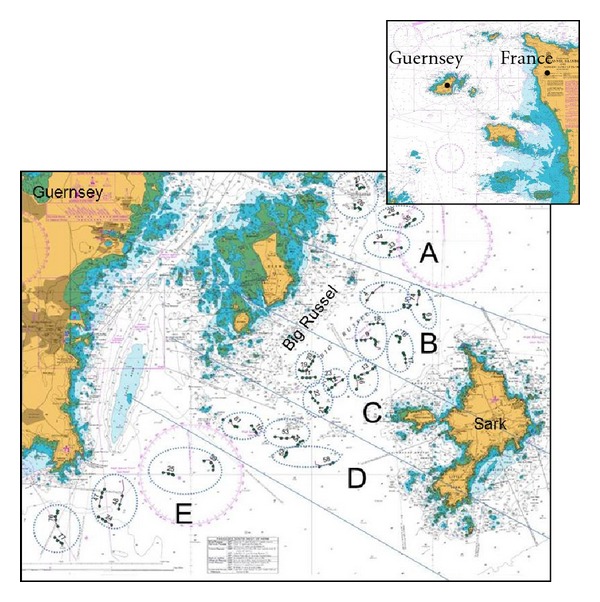
The Bailiwick of Guernsey off the north coast of France. The Big Russel channel is on the eastern side of Guernsey. The channel was divided into Locations (A, B, C, D, and E) and Areas (dotted lines), which comprise 2 or 3 sites (black filled in circles).

The islands of Guernsey (termed the Bailiwick) are situated in the bay of St Malo in the English Channel approximately 30 miles off the northern coast of France (Figure 1). The tidal currents around the Bailiwick are some of the strongest in the world, and the exposure to wave action from the Atlantic Ocean make this area a good prospective location to harness marine renewable energy [13]. Guernsey aims to generate 20% of its electricity from renewable sources by 2020 in line with EU targets [13] by locating tidal devices in “the Big Russell” (Figure 1), a channel where very little is known about the benthic assemblages. In order to predict the impact of future energy developments and allow managers to locate developments in areas of least impact, a benthic survey was undertaken.

The aim of the survey was to document the epibenthos in the Big Russel to provide a baseline of species composition in an area where tidal development may occur, and to identify suitable control areas for any future tidal development impact assessments. The survey also provided a reference list of species that can be used for future impact assessments for developers seeking consent to deploy devices on the sea bed. 

## 2. Methods

### 2.1. Study Site

Sites were selected across the Big Russel to include the parts which had been identified as potential locations for the development of tidal energy, and to identify suitable control areas for future impact assessment (Figure 1). Sites were also selected south of the Big Russell, as this was thought most likely to provide suitable controls away from those areas proposed for development in the main channel.

**Figure 2:**
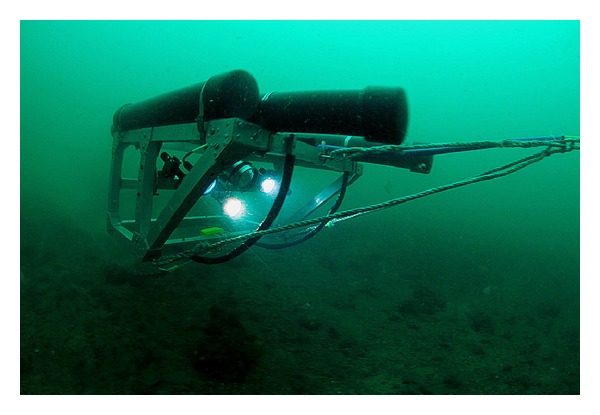
The towed flying array mounted with high definition video.

To enable quantitative comparison of the species assemblages throughout the north east to south west of the channel, transects were distributed across 5 “locations” A–E (Figure 1). To examine the small scale variability of the species assemblages within each “location” and increase our ability to estimate the species variability for any given part of the Big Russel, two to three transects (sites) were sampled per area. For each site, a transect was filmed for approximately 200 m with a field of view of 0.5 m width (Figure 2). A total of 36 transects were selected for video analysis based on the clarity of the footage. 

### 2.2. Field Sampling

Transects were videoed in September 2010 from a 15 m fishing trawler. The survey employed a method of filming the seabed using a High Definition (HD) video camera mounted on a towed “flying array” described by Sheehan et al. [6] (Figure 2). The flying array is an aluminium sled that floats above the seabed, which makes it suitable for sampling epibenthos over variable seabed relief. A piece of chain is used to control height above the seabed and a drop weight is attached to the tow rope to provide extra stability and minimize the effect of the pitch and roll of the boat.

The system comprised an HD video camera (Surveyor-HD-J12 colour zoom titanium camera, 6000 m depth rated, 720p: resolution 1280 × 720 (0.9 megapixels)) positioned at a 45° angle to the seabed, three LED lights, mounted either side and below the camera, and two green laser pointers. The two laser pointers were mounted on the frame either side of the camera at a fixed distance apart to allow calibration of the field of view during video analysis. The flying array was deployed over the stern of the boat and an umbilical connected the camera to the surface control unit allowing control of the camera focus, zoom, and light intensity [6]. The boat was carefully controlled and towed the camera slowly (approx. 0.4 knots) over the seabed in up to 2.4 knots of tidal flow. 

This method is cost effective, allowing large areas to be surveyed rapidly (e.g., Stevens and Connolly, [14]). It also has minimal impact on the seabed, which is essential for studies where there is interest in documenting change over time as it avoids confounding the results with impacts resulting from the survey method. The use of HD video provides data of a high quality, and also a data archive for future use.

### 2.3. Video Analysis

Video footage was analysed in two stages. To enumerate the abundant/encrusting species, including sponges, hydroids and algae, frame grabs were extracted at 5 s intervals and overlaid with a digital quadrat (3Dive Frame Extractor Software). Frames were viewed and those that were not clear of obstruction, well focused and had the lasers within acceptable margins of the screen, were deleted (see Sheehan et al., [6] for details). Ten frame grabs were haphazardly selected from the video throughout the length of the transect and all taxa within the frame identified and counted. Taxa were identified to the highest taxonomic level. Taxonomically similar species, which could not be distinguished with confidence, were grouped (e.g., branching sponges, gobies, and hydroids). The area sampled was corrected for every frame based on the position of the laser dots, giving density units of ind·m^−2^. To quantify the infrequent/conspicuous species including crustaceans, soft corals, and sea stars, counts were made from the entire video transect. Species counts were determined by viewing the video and recording all identifiable taxa that passed within the “gate” made by the two laser pointers (see the species list in the Supplementary Material available online at http://dx.doi.org/10.1155/2013/906180).

### 2.4. Statistical Analyses

Permutational multivariate analysis of variance (PERMANOVA+ in the PRIMER v6 software package, [15]) was used to determine whether assemblages of organisms were different between locations and areas based on Bray Curtis similarity matrices [16]. PERMANOVA is robust to datasets with many zeros and allows testing interactions in multivariate data. It has significant advantages over conventional MANOVA in that it makes no assumptions about underlying data distributions and is robust to unbalanced designs [17]. All analyses were done twice; firstly the common/encrusting fauna quantified from the ten frame grabs were averaged to avoid pseudoreplication and to increase the precision at which the epibenthic assemblage could be quantified. Secondly, an analysis was done for the infrequent/conspicuous fauna that were quantified from the entire video tow.

To examine spatial differences between assemblages there were three factors: Location (A–E), Area (random and nested in Location), and Site (random and nested in Area). Significant differences were further examined using pairwise tests. SIMPER was used to explain which taxa contributed most to differences between assemblages [18].

Multivariate assemblage data were visualised using nonmetric multidimensional scaling (nMDS) ordinations, one for the abundant/encrusting species (frame grabs), and one for the infrequent/conspicuous fauna (video dataset).

Potential habitat/taxa associations were then visualised by plotting frame grab assemblage data averaged over site, coded by the dominant habitat type for each site on nMDS ordination. The densities of the ten most abundant taxa for the three dominant habitats were also summarised in a table.

## 3. Results

The benthic community in the Big Russel was clearly affected by strong tides as throughout the channel the sessile fauna were typically cropped and low lying, and fishes were often observed travelling backwards, or fighting to swim towards rocky overhangs, presumably, to escape the tidal currents.

The area surveyed ranged from sandy plains in Location A in the north east (site 28) to bedrock and rocky pinnacles in Locations C and D. The largest proportion of frames (36.34%) was rock, with 31.34% composed entirely of bedrock. Cobbles and boulders were the next most common habitats, occurring in 27.05% and 18.43% of frames, respectively, and 13.68% of the frames comprised combined habitat.

A total of 74 taxa were identified during the survey, 39 in the video transects, and 59 in the frame grabs (full species list in Supplementary Material, Table 1). The most abundant species identified in the video transects was dead man's fingers *Alcyonium digitatum* followed by ross coral *Pentapora fascialis*. The most common taxa in the frame grabs were hydroids (grouped), which were present in 87.5% of the frames, followed by turf (hydroids and bryozoans < 1 cm), which was present in 75.5% of the frames. Other species observed included ballan wrasse *Labrus bergylta*, common cuttlefish *Sepia officinalis*, spiny spider crab *Maja squinado*, red gurnard *Aspitrigla cuculus*, bloody henry sea star *Henricia oculata,* edible crab *Cancer pagurus*, jewel anemone *Corynactis viridis*, and edible sea urchin *Echinus esculentus*. In the north of Big Russel where it is sandy we also observed flatfishes such as brill *Scophthalmus rhombus*.

**Figure 3:**
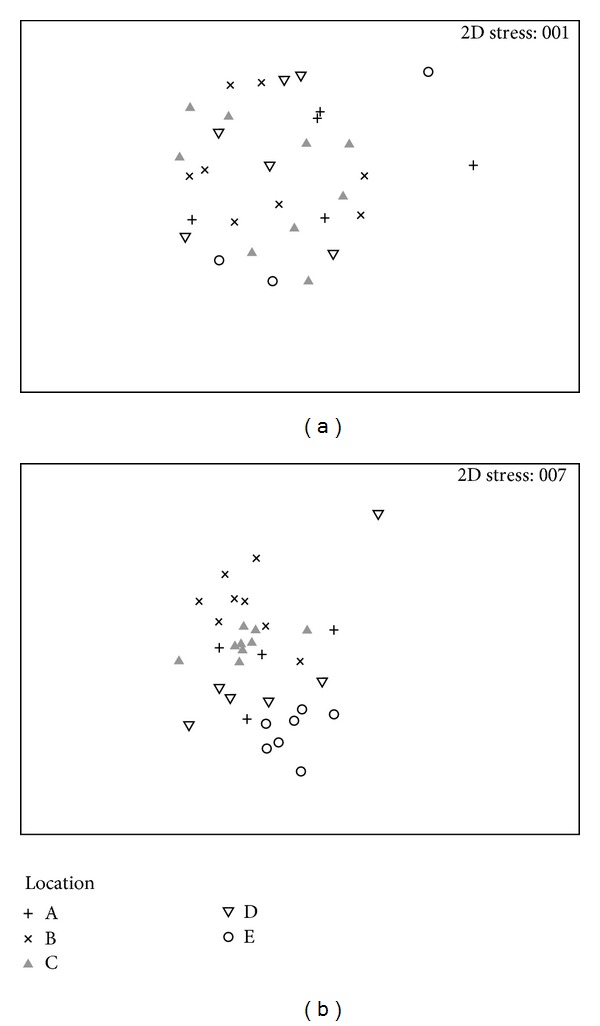
(a) nonmetric Multidimensional Scaling (nMDS) ordination of Bray-Curtis similarities of assemblage composition of the abundant/encrusting between Locations (A–E). (b) nMDS ordination of the Bray-Curtis similarities of assemblage composition of the infrequent/conspicuous fauna between Locations (A–E). Data were dispersion weighted and square root transformed.

The assemblage composition of benthic fauna in the Big Russel was highly variable. Locations were significantly different to each other for frame grab and video transect analyses (*P* < 0.05, Tables 1 and 2). Pairwise tests for the abundant/encrusting assemblage composition showed that Location A was not significantly different to any other Location. B and C were also not different to each other, suggesting that assemblages of the abundant and/encrusting fauna in the northern part of the channel were fairly similar. Conversely, locations in the southern end of the channel were significantly different to each other (*P* < 0.05) (Table 1) showing greater variability than in the northern end. The assemblage composition of the infrequent/conspicuous fauna was also similarly significantly different between locations (*P* < 0.05) (Figure 3(a)). 

The areas in each Location were not significantly different, showing that differences between species assemblages varied along the north east-south west gradient rather than between the sides of the channel. This has important implications for the future selection of control areas with regards to tidal energy impact assessment. Control areas will be best selected for across the channel rather than up or down stream of the devices. 

Across all Locations in the Big Russel, the most abundant sessile taxa identified from the frame grab analysis were hydroids (grouped), turf, and unidentified sponges. 

Prominent taxa in each location were red algae in Locations A, B, and C; bryozoan *Flustra foliacea* contributed towards the similarities between areas in Location B, keelworm *Pomatoceros triqueter* in Location D, and the bryozoans *Cellepora pumicosa* and *Pentapora fascialis *in Location E.

From the video analysis, the most frequently observed taxa of the infrequent/conspicuous species in the Big Russel were the spiny sea star *Marthasterias glacialis* and the bloody henry sea star *Henricia oculata*, which contributed towards the similarities between areas in all locations. Varying abundances of dead man's fingers *Alcyonium digitatum* and the crabs; the spiny spider crab *Maja squinado*, the velvet swimming crab *Necora puber,* and the edible crab *Cancer pagurus* all contributed to differences along the north east-south west gradient between locations (Table 3).

Despite the PERMANOVA results indicating differences between both the abundant/encrusting fauna and the infrequent/conspicuous fauna, the nMDS ordinations suggested that the pattern was different between the two groups (Figure 3). A clear gradient can be seen for the infrequent/conspicuous fauna that shows distinct grouping for each location, with locations situated next to their geographical neighbour. There are no discernible patterns, however, for the common/encrusting fauna.

### 3.1. Habitats in the Big Russel

The dominant habitat types identified were rock and boulders and cobbles. Sand was the dominant habitat type in some frame grabs in the north of the channel and is therefore included as a dominant habitat type despite being relatively rare throughout the rest of the Big Russel.

The habitat type supporting the greatest abundance of taxa was rock (50 taxa), but the mean abundance of individuals was greatest on the boulders and cobbles (74.33 individuals site^−1^). Frames dominated by sand were by comparison species poor (12 taxa and 11 individuals site^−1^). Some taxa were found to dominate across all habitat types but their abundance was greater in frames where rock, boulders, or cobbles were present (Table 4).

Epifauna were only present in sandy habitats when the frame contained hard substrata. With the exception of the sand eel *Ammodytes tobianus *the species present in sandy habitats were those associated with rocky substrata.

**Figure 4:**
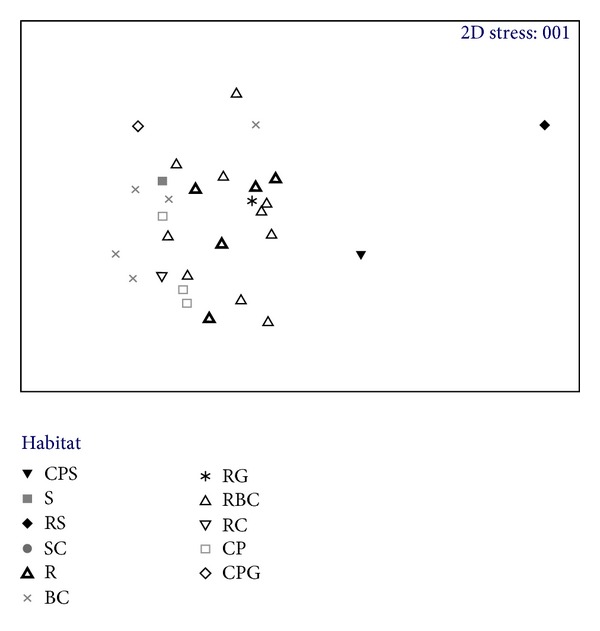
nonmetric Multidimensional Scaling (nMDS) ordination showing the similarities between abundant/encrusting species assemblages at different sites based on habitat type. Habitat type is the dominant type per tow calculated from the frame analysis (R (rock), B (boulders), C (cobbles), P (pebbles), G (gravel), and S (sand)).

The differences between species assemblage composition averaged over site from the frame grab data can be partially explained by habitat type. Sites where boulders and cobbles dominated the frames show some aggregation of species assemblage composition. Sites where rock dominated the frames also show similarities between species assemblage composition. Site 26 (Location A), which was dominated by rock and sand, was dissimilar to all other sites (black diamond on the left side of the ordination, Figure 4).

## 4. Discussion

The extent of the benthic features in the high tidal energy site, the Big Russell, was successfully recorded. Using the “flying array” a range of epifauna were enumerated from flatfishes on the sandy plains in the north of the channel to crustaceans and bryozoans on the heterogeneous reef habitat in the main channel. Overall, 74 epifaunal taxa were counted.

Areas which were composed entirely of bedrock were those with the greatest number of species. Cobbles and pebbles supported the second greatest abundance of species followed by boulders and cobbles. In the sandy habitat (e.g., Location A), although few mobile fauna such as flatfish were recorded, supplementary sampling would be required to fully assess the habitat as the greatest abundance of fauna in sedimentary habitats occurs below the surface, “infauna” [19], which the video does not sample. Quantification of infauna would require dredges or a grab to take physical samples [20].

Some taxa such as turf and hydroids were found to dominate across all hard substrate habitat types. Due to the tide swept environment, fauna associated with the hard habitat types were characterised by species such as encrusting sponges, dead man's fingers *Alcyonium digitatum*, ross coral *Pentapora fascialis,* and hornwrack *Flustra foliacea* which grow close to the substratum.

The Guernsey Regional Environmental Assessment (REA) identified that seagrass beds and maerl beds were the priority habitats for protection in Guernsey. Neither of which were identified during this study. Furthermore, no UK Biodiversity Action Plan (BAP) species have been identified here. It is important to note, however, that this method does not sample all benthic fauna. Species such as the BAP species cup coral *Leptopsammia pruvoti* are commonly found under overhangs and in small crevices [21] and are therefore not likely to be identified through a study using a towed camera that flies above the benthos.

Based on this survey, it is difficult to assess the implications of the placement of future tidal devices without knowledge of the type and size of the devices. Observations of the extremely heterogenous seabed comprising sandy patches, cobbles, boulders, and rocky pinnacles in an area known for extreme tides and waves suggest that the benthic faunal assemblages are already living in a diverse and hostile environment. Deployment of devices in the north where there are sandy patches would introduce additional hard habitat for epifauna to colonise [22]. Construction throughout the channel may cause localised disturbance to fauna, but ultimately, devices are likely to act as artificial reefs like other anthropogenic structures [23–25] providing increased habitat complexity that benthic mobile fauna such as crustaceans could use as a refuge [26] and fishes may use to escape tidal currents in this high energy environment. The risks associated with the devices such as collision are not likely to affect those benthic organisms discussed here, but should be considered for larger pelagic species [27].

Deployment of marine renewable devices not only introduces impacts to the benthos but is also known to relieve other human impacts such as the effects of trawling and dredging [5, 9, 28, 29]. However, after observing the seabed in the Big Russel it was clear that fishing using static gear, in particular pots, was most common rather than the use of more destructive towed gears. The rocky pinnacles and reefs that were observed provide the perfect complex habitat for benthic fauna but would certainly snag and break most towed fishing gears.

Location E had been suggested as a potential control area away from the likely area for tidal development by the Guernsey Renewable Energy Team. The assemblage of organisms found in Location E was statistically different to all the other Locations and so it would not be comparable to locations in the main channel. Despite the PERMANOVA results indicating differences between both the abundant/encrusting fauna and the infrequent/conspicuous fauna, the nMDS ordinations suggested that the pattern was different between the two groups. A clear latitudinal gradient was seen for the infrequent/conspicuous fauna that shows distinct grouping within each location, which are separated and situated next to their geographical neighbour on the nMDS. There were no discernible patterns, however, for the common/encrusting fauna. Unlike the infrequent/conspicuous taxa, and as a result of using flying HD video, many of the abundant/encrusting taxa could not be identified to species. For example, “turf,” “hydroids,” and “sponges” and so any potential existing differences that may exist at the species level through the channel may not occur at the observed lower level of taxonomic resolution. To resolve this problem, future analyses may be best combined across video analysis methods to give an estimate of overall assemblage. Future impact assessments can also use this study to preselect a subset of indicator species that represent the response of different groups of organisms that share life history traits [30]. An example of a life history trait could be “Recoverability from disturbance” where dead man's fingers *Alcyonium digitatum* could be used as an indicator species for those with “Low recoverability,” edible crabs *Cancer pagurus* have “Medium recoverability,” and the great scallop *Pecten maximus* are quick to recover from disturbance and so represent species with relatively “High recoverability” [30]. 

The species assemblage changed over the latitudinal gradient, and so depending on the location of future developments, the most suitable, comparable un-impacted controls would likely be found in a similar latitude to the development. 

This study has provided a baseline assessment of the epibenthos of the Big Russel. The results can be used to inform the optimal siting of future tidal energy devices in the channel and as a baseline for future impact assessment.

## Supplementary Material

Table 1 contains a list of all taxa enumerated from the video analysis during the epibenthic assessment of the renewable tidal energy site in Guernsey, UK.

## Figures and Tables

**Table:** (a)

Source	df	MS	Pseudo-F	*P* (perm)
Location Lo	4	2246.6	1.9995	**0.0029**
Area Ar(Lo)	11	1125.4	1.2587	0.0825
Site (Ar(Lo))	16	894.08	No test	

Total	31			

**Table:** (b)

Location pairings	P (perm)
A and B	0.1719
A and C	0.1233
A and D	0.4978
A and E	0.4014
B and C	0.1127
B and D	**0.0270**
B and E	**0.0107**
C and D	**0.0218**
C and E	**0.0047**
D and E	**0.0470**

**Table:** (a)

Source	df	MS	Pseudo-F	*P* (perm)
Location Lo	4	5036.5	2.8667	**0.0006**
Area Ar(Lo)	12	1725.4	2.1951	**0.0001**
Site Si(Ar(Lo))	19	786.02	No test	

Total	35			

**Table:** (b)

Location pairings	*P* (perm)
A and B	0.0565
A and C	**0.0146**
A and D	0.4946
A and E	0.2627
B and C	**0.0343**
B and D	**0.0298**
B and E	**0.0092**
C and D	**0.0145**
C and E	**0.0032**
D and E	0.0501

**Table:** (a)

Frames	Av. abund.	Contrib. percentage
Location A		

Grouped hydroids	0.63	16.19
Turf algae	0.65	14.62
Encrusting sponge 4	0.5	11.54
Red algae	0.42	10.97
Encrusting sponge 1	0.52	9.78
Encrusting sponge 2	0.48	7.76

Location B		

Grouped hydroids	0.93	20.14
Turf algae	0.68	13.2
Encrusting sponge 1	0.6	11.51
Red algae	0.61	11.02
*Flustra foliacea *	0.55	10.37
Encrusting algae 2	0.48	9.18

Location C		

Grouped hydroids	0.97	21.31
Turf algae	0.77	14.53
Red algae	0.48	9.26
Encrusting sponge 4	0.45	8.57
Encrusting sponge 1	0.46	7.5
Encrusting sponge 2	0.38	5.9

Location D		

Grouped hydroids	0.92	20.81
Turf algae	0.82	17.61
*Pomatoceros triqueter *	0.7	12.96
Encrusting sponge 2	0.52	8.31
Encrusting sponge 1	0.5	6.78
*Nemertesia antennina *	0.38	5.2

Location E		

Turf algae	0.8	16.76
Encrusting sponge 4	0.8	15.86
Hydroids (grouped)	0.73	15.15
*Cellepora pumicosa *	0.73	13.6
Encrusting sponge 1	0.7	12.53
*Pentapora foliacea *	0.67	11.85

**Table:** (b)

Video transects	Av. abund.	Contrib. percentage
Location A		

*Marthasterias glacialis *	1.45	24.71
*Alcyonium digitatum *	0.8	13.37
*Henricia oculata *	0.81	9.7
*Ammodytes tobianus *	0.34	8.8
*Pentapora foliacea *	0.63	8.68
*Cancer pagurus *	0.52	6.22

Location B		

*Marthasterias glacialis *	1.66	17.76
*Henricia oculata *	1.55	13.8
*Cliona celata *	1.19	12.4
*Cancer pagurus *	1.32	11.1
*Ctenolabrus rupestris *	1.21	10.92
*Necora puber *	1.05	8.37

Location C		

*Marthasterias glacialis *	1.93	15.88
*Henricia oculata *	1.72	15.04
*Polymastia boletiformis *	1.25	10
*Alcyonium digitatum *	1.15	8.81
*Cancer pagurus *	0.97	8.23
Branching sponge 1	1.05	8.05

Location D		

*Marthasterias glacialis *	1.18	18.36
*Pentapora foliacea *	1.39	14.06
*Henricia oculata *	1.01	13.35
Branching sponge 2	1.45	12.06
*Polymastia boletiformis *	1.1	8.88
Branching sponge 4	0.95	6.67

Location E		

Branching sponge 2	1.3	15.75
*Marthasterias glacialis *	1.41	15.59
*Henricia oculata *	0.99	11.54
*Pentapora foliacea *	1.04	10.41
*Alcyonium digitatum *	0.65	7.02
*Maja squinado *	0.78	5.85

**Table 4:** The ten taxa from frame grab analysis with the greatest abundance where rock, boulders and cobbles, and sand were the dominant habitat type. Data are percentage of frames containing each taxa for each habitat type (%). Gravel and pebbles were excluded as they did not dominate the habitat in any frame.

Rock		Boulders and Cobbles		Sand	
Taxa	Percentage	Taxa	Percentage	Taxa	Percentage
Turf	72.17	Hydroids (grouped)	72.73	Hydroids (grouped)	20.00
Hydroids (grouped)	65.41	Turf	65.16	Red algae	20.00
Encrusting sponge 1	61.77	*Pomatoceros triqueter *	50.19	Encrusting sponge 4	15.00
*Pomatoceros triqueter *	51.77	Encrusting sponge 4	31.32	Turf	15.00
Encrusting sponge 2	50.10	*Flustra foliacea *	28.05	*Alcyonium digitatum *	5.00
Encrusting sponge 4	33.54	Encrusting sponge 1	26.40	*Ammodytes tobianus *	5.00
Encrusting sponge 3	33.33	Encrusting sponge 2	25.58	*Calliostoma zizyphinum *	5.00
*Nemertesia antennina *	30.00	*Nemertesia antennina *	24.63	*Dendrodoa grossularia *	5.00
*Alcyonium digitatum *	25.20	*Pentapora fascialis *	20.15	*Halecium halecinum *	5.00
Red algae	23.74	*Alcyonium digitatum *	19.71	*Nemertesia antennina *	5.00

## References

[1] Pelc R, Fujita RM (2002). Renewable energy from the ocean. *Marine Policy*.

[2] Cada G, Ahlgrimm J, Bahleda M (2007). Potential impacts of hydrokinetic and wave energy conversion technologies on aquatic environments. *Fisheries*.

[3] Carbon Trust (2006). *Future Marine Energy. Results of the Marine Energy Challenge: Cost Competitiveness and Growth of Wave and Tidal Stream Energy*.

[4] Kerr D (2007). Marine energy. *Philosophical Transactions of the Royal Society A*.

[5] Inger R, Attrill MJ, Bearhop S (2009). Marine renewable energy: potential benefits to biodiversity? An urgent call for research. *Journal of Applied Ecology*.

[6] Sheehan EV, Stevens TF, Attrill MJ (2010). A quantitative, non-destructive methodology for habitat characterisation and benthic monitoring at offshore renewable energy developments. *PLoS ONE*.

[7] Gill AB (2005). Offshore renewable energy: ecological implications of generating electricity in the coastal zone. *Journal of Applied Ecology*.

[8] Linley EAS, Wilding TA, Black K, Hawkins AJS, Mangi S (2007). Review of the reef effects of offshore wind farm structures and their potential for enhancement and mitigation. *Report to the Department for Business, Enterprise and Regulatory Reform*.

[9] Kaiser MJ, Clarke KR, Hinz H, Austen MCV, Somerfield PJ, Karakassis I (2006). Global analysis of response and recovery of benthic biota to fishing. *Marine Ecology Progress Series*.

[10] Witt MJ, Sheehan EV, Bearhop S (2012). Assessing wave energy effects on biodiversity: the Wave Hub experience. *Philosophical Transactions of the Royal Society A*.

[11] Grecian WJ, Inger R, Attrill MJ (2010). Potential impacts of wave-powered marine renewable energy installations on marine birds. *IBIS*.

[12] Shields MA, Dillon LJ, Woolf DK, Ford AT (2009). Strategic priorities for assessing ecological impacts of marine renewable energy devices in the Pentland Firth (Scotland, UK). *Marine Policy*.

[13] Guernsey Renewable Energy Team Regional Environmental Assessment of Marine Energy. http://www.guernseyrenewableenergy.com/documents/managed/REA%20Final/CH%201%20-%203.pdf.

[14] Stevens T, Connolly RM (2005). Local-scale mapping of benthic habitats to assess representation in a marine protected area. *Marine and Freshwater Research*.

[15] Anderson MJ (2001). A new method for non-parametric multivariate analysis of variance. *Austral Ecology*.

[16] Bray JR, Curtis JT (1957). An ordination of upland forest communities of southern Wisconsin. *Ecological Monographs*.

[17] Walters K, Coen LD (2006). A comparison of statistical approaches to analyzing community convergence between natural and constructed oyster reefs. *Journal of Experimental Marine Biology and Ecology*.

[18] Clarke KR, Warwick RM (2001). *Change in Marine Communities: An Approach to Statistical Analysis and Interpretation*.

[19] Beukema JJ (1976). Biomass and species richness of the macro-benthic animals living on the tidal flats of the Dutch Wadden Sea. *Netherlands Journal of Sea Research*.

[20] Eleftheriou A, Robertson MR (1992). The effects of experimental scallop dredging on the fauna and physical environment of a shallow sandy community. *Netherlands Journal of Sea Research*.

[21] Goffredo S, Caroselli E, Pignotti E, Mattioli G, Zaccanti F (2007). Variation in biometry and population density of solitary corals with solar radiation and sea surface temperature in the Mediterranean Sea. *Marine Biology*.

[22] Petersen JK, Malm T (2006). Offshore windmill farms: threats to or possibilities for the marine environment. *Ambio*.

[23] Rilov G, Benayahu Y (2002). Rehabilitation of coral reef-fish communities: the importance of artificial-reef relief to recruitment rates. *Bulletin of Marine Science*.

[24] Love MS, Caselle J, Snook L (1999). Fish assemblages on mussel mounds surrounding seven oil platforms in the Santa Barbara Channel and Santa Maria Basin. *Bulletin of Marine Science*.

[25] Helvey M (2002). Are southern California oil and gas platforms essential fish habitat?. *ICES Journal of Marine Science*.

[26] Langhamer O, Wilhelmsson D (2009). Colonisation of fish and crabs of wave energy foundations and the effects of manufactured holes—a field experiment. *Marine Environmental Research*.

[27] Wilson B, Batty RS, Daunt F, Carter C (2007). Collision risks between marine renewable energy devices and mammals, fish and diving birds. *Report to the Scottish Executive*.

[28] Roberts CM, Hawkins JP, Gell FR (2005). The role of marine reserves in achieving sustainable fisheries. *Philosophical Transactions of the Royal Society B*.

[29] Fayram AH, de Risi A (2007). The potential compatibility of offshore wind power and fisheries: an example using bluefin tuna in the Adriatic Sea. *Ocean and Coastal Management*.

[30] Jackson EL, Langmead O, Barnes M, Tyler-Walters H, Hiscock K (2008). Identification of indicator species to represent the full range of benthic life history strategies for Lyme Bay and the consideration of the wider application for monitoring of Marine Protected Areas. *Report to the Department of Environment, Food and Rural Affairs from the Marine Life Information Network (MarLIN)*.

