# Supplementary material for: Epibenthic Assessment of a Renewable Tidal Energy Site
Source: ScientificWorldJournal. 2013 Feb 6;2013:906180. doi: 10.1155/2013/906180 (PMC3580905; doi:10.1155/2013/906180)
Supplement: Supplementary file 1 — Table 1 contains a list of all taxa enumerated from the video analysis during the epibenthic assessment of the renewable tidal energy site in Guernsey, UK. [file 906180.f1.docx]

**Supplementary material**

Supplementary material. Table 1.

| **Taxa** | **Common name** |
| --- | --- |
| *Aequipecten opercularis* | Queen scallop |
| *Alcyonidium diaphanum* | Sea chervil |
| *Alcyonium digitatum* | Dead man's fingers |
| *Ammodytes tobianus* | Sand eel |
| *Anseropoda placenta* | Goose foot starfish |
| *Aspitrigla cuculus* | Red Gurnard |
| *Botryllus schlosseri* | Star ascidian |
| Branching sponge 1 | A branching sponge |
| Branching sponge 2 | A branching sponge |
| Branching sponge 3 | A branching sponge |
| Branching sponge 4 | A branching sponge |
| *Callionymus lyra* | Common Dragonet |
| *Calliostoma zizyphinum* | Painted topshell |
| *Cancer pagurus* | Edible crab |
| *Caryophyllia smithii* | Devon cup coral |
| *Cellaria fistulosa* | A bryozoan |
| *Cellepora pumicosa* | A bryozoan |
| *Ciona intestinalis* | A sea squirt |
| *Cliona celata* | Boring sponge |
| *Conger conger* | Conger eel |
| *Corynactis viridis* | Jewel anemone |
| *Ctenolabrus rupestris* | Goldsinny wrasse |
| *Dendrodoa grossularia* | Baked bean ascidian |
| *Dercitus bucklandi* | An encrusting sponge |
| *Echinus esculentus* | Edible sea urchin |
| *Eunicella verrucosa* | Pink sea fan |
| *Flustra foliacea* | Hornwrack |
| *Galathea dispersa* | A squat lobster |
| Goby | Gobies (grouped) |
| *Gymnangium montagui* | Yellow feathers |
| *Halichondria panicea* | Breadcrumb sponge |
| *Hemimycale columella* | An encrusting sponge |
| *Henricia oculata* | Bloody henry |
| *Holothuria forskali* | Cotton spinner |
| Grouped hydroids | Hydroids (grouped) |
| *Inachus spp.* | Spider crabs |
| *Labrus bergylta* | Ballan wrasse |
| *Labrus mixtus* | Cuckoo wrasse |
| *Lipophrys pholis* | Shanny |
| *Luidia cilaris* | A starfish |
| *Macropodia spp.* | Spider crabs |
| *Maja squinado* | Spiny spider crab |
| *Marthasterias glacialis* | Spiny starfish |
| *Necora puber* | Velvet swimming crab |
| *Nemertesia antennina* | Sea beard |
| *Nemertesia ramosa* | A hydroid |
| *Ophiura ophiura* | A brittlestar |
| *Pachymatisma johnstonia* | A sponge |
| *Parablennius gattorugine* | Tompot Blenny |
| *Pecten maximus* | Great scallop |
| *Pentapora foliacea* | Ross coral |
| *Pholis gunnellus* | Butterfish |
| *Polymastia boletiformis* | A sponge |
| *Pomatoceros triqueter* | Keelworm |
| *Raja clavata* | Thornback ray |
| Red algae | Red algae (grouped) |
| *Sabella pavonina* | Peacock worm |
| *Sagartia elegans* | A sea anemone |
| *Sepia officinalis* | Common cuttlefish |
| *Serpula vermicularis* | A tubeworm |
| Encrusting sponge 1 | An encrusting sponge |
| Encrusting sponge 2 | An encrusting sponge |
| Encrusting sponge 3 | An encrusting sponge |
| Encrusting sponge 4 | An encrusting sponge |
| Encrusting sponge 5 | An encrusting sponge |
| Encrusting sponge 6 | An encrusting sponge |
| Massive sponge 1 | A massive sponge |
| Massive sponge 2 | A massive sponge |
| *Suberites domuncula* | Sea orange, sulphur sponge |
| *Trisopterus luscus* | Pouting |
| *Trisopterus minutus* | Poor-cod |
| *Tubularia indivisa* | A hydroid |
| Turf | Turf algae |
| *Zeugopterus punctatus* | Topknot |
